# Immature central tumor tertiary lymphoid structures are associated with better prognosis in non-small cell lung cancer

**DOI:** 10.1186/s12890-024-02970-6

**Published:** 2024-03-26

**Authors:** Deng Xiaoxu, Xu Min, Cao Chengcheng

**Affiliations:** 1 Pathology Department, Shengjing Hospital of China Medical University, Shenyang Liaoning, China; 2 Department of Thoracic Surgery, Liaoning Cancer Hospital&Institution, Shenyang Liaoning, China

**Keywords:** Non-small cell lung cancer, Tertiary lymphoid structure, CD8, Prognosis

## Abstract

**Background & aims:**

Tertiary lymphoid structures (TLSs) are predictive biomarkers of favorable clinical outcomes and immunotherapy response in several solid malignancies, including non-small cell lung cancer (NSCLC). However, the relationship between TLSs and NSCLC prognosis has not been eludicated from the aspects of location, density, and maturity. This study aimed to investigate the clinicopathological and prognostic significance of TLSs in NSCLC.

**Methods:**

A collection of 151 resected pulmonary nodules in patients with NSCLC was retrospectively analyzed. Two experienced pathologists reviewed hematoxylin-eosin (H&E) slides and assessed TLS scores at different anatomic subregions. Then, we analyzed their correlation with clinicopathologic parameters and CD8 staining intensity and assessed multiple clinicopathological factors affecting patient prognosis.

**Results:**

CD8 expression was correlated with total (TLS-CT) (*P* = 0.000), aggregates (Agg) (TLS-CT) (*P* = 0.001), follicles (FOL)-I (TLS-CT) (*P* = 0.025), and TLS(overall) (*P* = 0.013). TLS scores in the central tumor (CT) and invasion margin (IM) areas were negatively correlated with distant metastasis and Union for International Cancer Control (UICC) stage in NSCLC patients, while TLS score in the CT area was positively correlated with CD8 expression. TLS (overall), Agg (TLS-CT), and FOL-I (TLS-CT) were positively correlated with distant metastasis, UICC stage, and CD8 expression in NSCLC patients. Agg (TLS-IM) was positively correlated with distant metastasis and UICC stage. FOL-I (TLS-IM) was positively correlated with UICC stage. FOL-II (TLS-IM) was positively correlated with distant metastasis (*P* < 0.05). Multivariate Cox regression analysis showed that unfavorable independent prognostic factors were associated with metastasis status and UICC stage. Independent prognostic factors with protective effects included Agg (TLS-CT), FOL-I (TLS-CT), total (TLS-CT), and overall TLS (*P* < 0.05).

**Conclusion:**

Histological score assessment of H&E sections of Agg (TLS-CT), FOL-I (TLS-CT), total (TLS-CT), and overall TLS levels in NSCLC has prognostic value.

## Introduction

The cancer statistics of China and the United States in 2022 ranked the incidence and mortality rate of lung cancer patients [1]. In the United States, the incidence of lung cancer is second only to breast cancer, and the mortality rate is first. Lung cancer has become one of the important causes of tumor-related death. From the pathological perspective, lung cancer can be roughly divided into non-small cell lung cancer (NSCLC) and small cell lung cancer (SCLC), among which NSCLC accounts for about 80–85%, including adenocarcinoma, squamous cell carcinoma, and other histological subtypes [2]. Apar et al. [3] reported that the 5-year survival rate of NSCLC was only 26.4%.

Tertiary lymphoid structures (TLSs) are named after the primary and secondary lymphatic organs (peripheral immune organs or peripheral lymphatic organs which includes the spleen, lymph nodes, pharyngeal tonsils, appendix, and lymph nodules and lymph tissue distributed throughout the body). Primary lymphatic organs mainly include the thymus and bone marrow. Secondary lymphatic organs include the spleen, lymph nodes, pharyngeal tonsils, appendix, and lymph nodules, as well as the lymph tissue distributed throughout the body. Lymphoid tissue associated with immunity, including mucosa-associated lymphoid tissue and skin-associated lymphoid tissue, is known as TLS. TLS is defined as an ectopic lymphaden-like structure in the tumor, which may be accompanied by germinal center formation, mainly including T cells, B cells, dendritic cells, and high endothelial vein [4, 5]. The purpose of this study was to observe TLS formation in NSCLC tissues and analyze its relationship with clinicopathologic features and patient prognosis. To determine whether TLSs in the tumor microenvironment are beneficial or harmful to patients, analyzing their location, density, and maturity is necessary [6].

## Materials and methods

### Study data

The study included 151 patients with NSCLC who underwent surgical treatment at Shengjing Hospital Affiliated to China Medical University between January 2013 and December 2015. Inclusion criteria were as follows: (1) patients diagnosed with NSCLC using histopathology; (2) patients first treated in our hospital, who had not received radiotherapy, chemotherapy, or anti-inflammatory therapy before surgery; and (3) patients with complete clinicopathological and postoperative follow-up data. The tumor clinical and pathological staging system was based on the 8th edition of Union for International Cancer Control (UICC)/American Joint Committee on Cancer. All patients included in this study provided written informed consent. The study and its protocols were approved by the Research Ethics Committee of Shengjing Hospital Affiliated with China Medical University.

### Approval number: 2022PS946K

#### Research methods

##### Clinical data collection

The basic data of patients at admission, including gender, age, and smoking status, were collected through the pathological diagnosis and hospital management systems. Patient clinicopathological characteristics, including the pathological type, differentiation degree, clinical stage, and tumor metastasis, were recorded.

##### Hematoxylin-eosin (H&E) staining and TLS score counting

All tumor tissues were fixed with 10% neutral formalin and made into wax blocks, which were successively sliced to have 5-µm thickness for H&E staining. The staining results were examined by two independent pathologists (DXX & CCC) in the department of pathology of our hospital. All pathological data gathered were blinded to the clinical diagnosis. For the location perspective, the tumor areas were divided into central tumor (CT) and invasive margin (IM, within 500 μm width on both sides of the tumor), and TLS scores were obtained for the two areas. With respect to density, the TLS percentage was evaluated in the tumor CT and IM areas. According to their *maturity* stages, TLSs was classified as lymphoid aggregates (Agg) and lymphoid follicles (FOL). FOL was further subdivided into FOL-I (lymphoid follicles without germinal centers) and FOL-II (with germinal centers) [7, 8].

##### Immunohistochemical staining and results

**Fig. 1:**
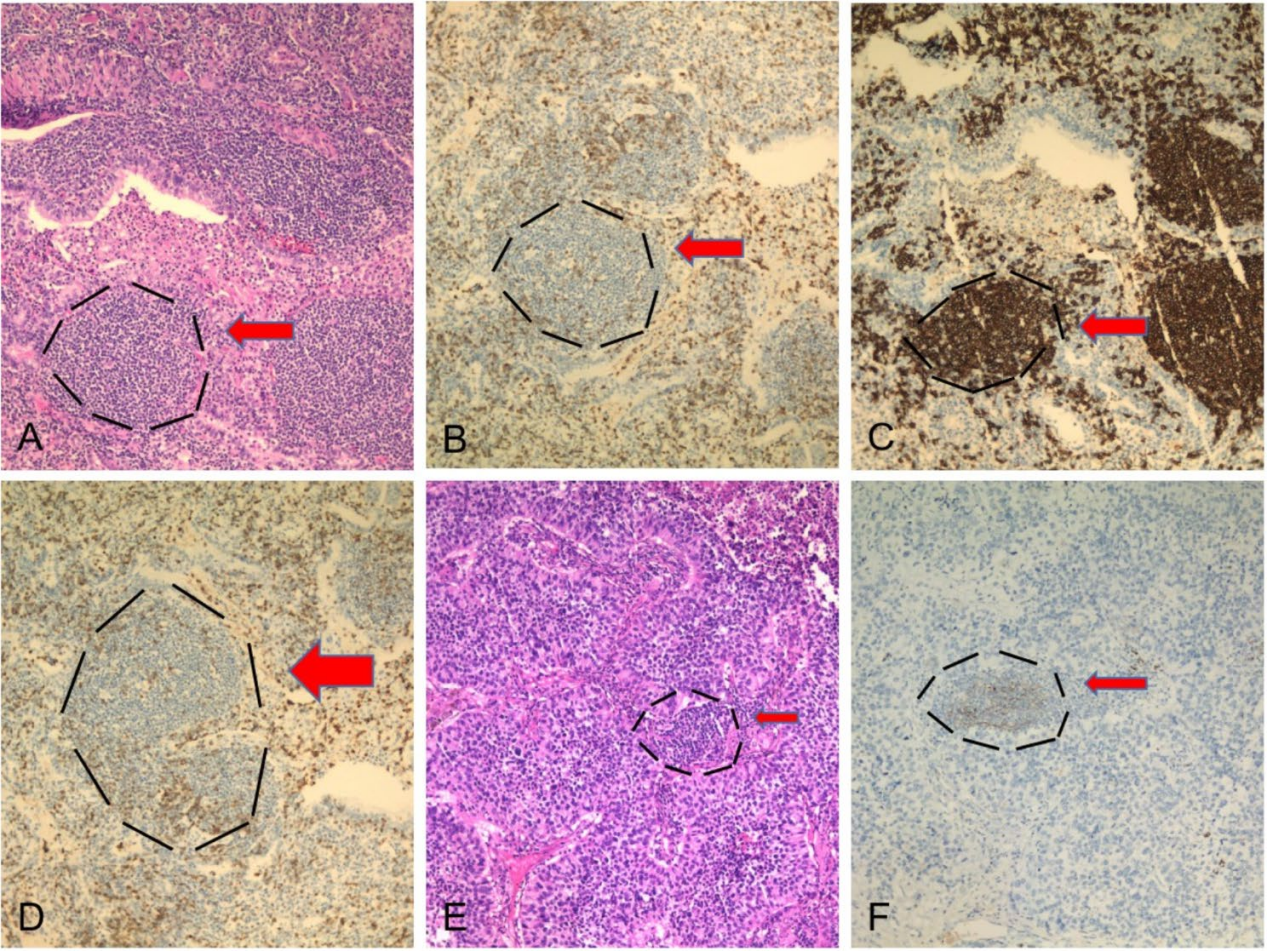
Characterization of NSCLC-associated TLS. (**A**) Representative H&E staining images of NSCLC tissue show TLSs in the invasive margin (IM) tissue. The ectopic lymphaden-like structure in the tumor may be accompanied by germinal center formation, which mainly includes T cells, B cells, dendritic cells, and high endothelial vein. (**B**) CD20 staining shows B cells. (**C**) CD3 staining shows T cells. (**D**) CD31 staining shows blood vessels and high endothelial veins. (**E**) Representative H&E staining images of NSCLC tissue show TLSs in the CT tissue. (**F**) CD23 staining shows dendritic cells(x100)

All specimens were routinely paraffin-embedded and sliced, and immunohistochemical staining was performed. Mouse anti-human CD8 antibody (Maixin Company) was used, and ready-made immunohistochemical and DAB kits (Maixin Company) were used according to the manufacturer’s instructions. The cell membrane of interstitial lymphocytes with CD8-positive staining was brown. CD8 staining criteria: semi-quantitative analysis was performed according to the percentage of positive cells; 10% was set as the cutoff point for dividing patients into high and low expression groups [7]. In cases wherein distinction was difficult, slides were additionally treated with anti-CD20, anti-CD3, anti-CD23, and anti-CD31 antibodies (all Ready-to-Use, Roche). These antibodies were employed to verify the presence of TLS using B cells, T cells, dendritic cells, and high endothelial veins, respectively (Fig. 1).

##### Follow-up

The postoperative survival of the patients was followed up via telephone and outpatient return visits to obtain the overall survival (OS). The date of last follow-up, which refers to the time elapsed from the date of surgery to the time of death or the end of follow-up, was May 1st, 2022. Patients who refused follow-up or could not be contacted for various reasons were considered lost to follow-up.

### Statistical method

SPSS 26.0 statistical software was used for statistical processing. The χ^2^ test was used to analyze the correlation between TLS, clinicopathological features, and CD8 expression. Kaplan–Meier survival analysis and a Cox regression model were used to analyze the relationship between TLS, clinicopathological features, and patient survival time. Differences with *P* < 0.05 were considered statistically significant.

## Results

### Correlation analysis between TLS and clinicopathologic features in the CT and IM areas of NSCLC

The average TLS percentage in the CT area (CTtotal) of 151 NSCLC patients was 7.5%. The average percentage of TLS in the IM area (IMtotal) was 3.6%. The data were divided into the CTtotal-low (88 cases, 58.3%), CTtotal-high (63 cases, 41.7%), IMtotal-low (99 cases, 65.6%), and IMtotal-high (52 cases, 34.4%) groups according to the median TLS scores in CT area and IM area. Among the 151 patients with NSCLC, 67 were males, and 84 were females. The age ranged from 38 to 78 years, with an average age of 61.0 years. These data revealed that high TLS levels in the CT and IM areas were associated with no distant metastasis and earlier UICC stage in NSCLC patients. Simultaneously, high TLS levels in the CT area were positively correlated with CD8 expression (*P* < 0.05, Table 1).

**Table 1:** Correlation between TLS, clinicopathological features, and CD8 staining in patients with NSCLC

Clinicopathological features	Cases	CTtotal*				IMtotal*			
		Low	High	χ^2^	P	Low	High	χ^2^	P
**Gender**				0.000	0.988			1.833	0.176
Female	84	49	35			59	25		
Male	67	39	28			40	27		
**Age**				0.057	0.811			0.205	0.650
< 60	63	36	27			40	23		
≥ 60	88	52	36			59	29		
**Smoking status**				6.580	0.010			0.618	0.432
Non-smoker	87	57	30			57	30		
Smoker	51	22	29			30	21		
**Differentiation**				3.403	0.065			0.089	0.765
Higher differentiation	99	53	46			65	34		
Middle-low differentiation	35	25	10			22	13		
**Histological type**				0.302	0.583			0.147	0.702
Adenocarcinoma	141	83	58			93	48		
Non-adenocarcinoma	10	5	5			6	4		
**Metastatic status**				17.381	0.000			10.959	0.001
Non-metastasis	56	20	36			27	29		
Metastasis	92	65	27			69	23		
**UICC stage**				25.236	0.000			17.868	0.001
I	47	26	21			7	40		
II	14	12	2			3	11		
III	57	49	8			19	38		
IV	32	31	1			18	14		
**CD8**				25.795	0.000			2.986	0.084
Negative	59	49	10			44	15		
Positive	84	34	50			51	33		

^*^CTtotal:the total TLS percentage of all maturity in the central tumor area

^*^IMtotal: the total TLS percentage of all maturity in the invasive margin area

*Correlation between different maturity and location of NSCLC-associated TLS and clinicopathologic features in CT and IM areas*.

**Fig. 2:**
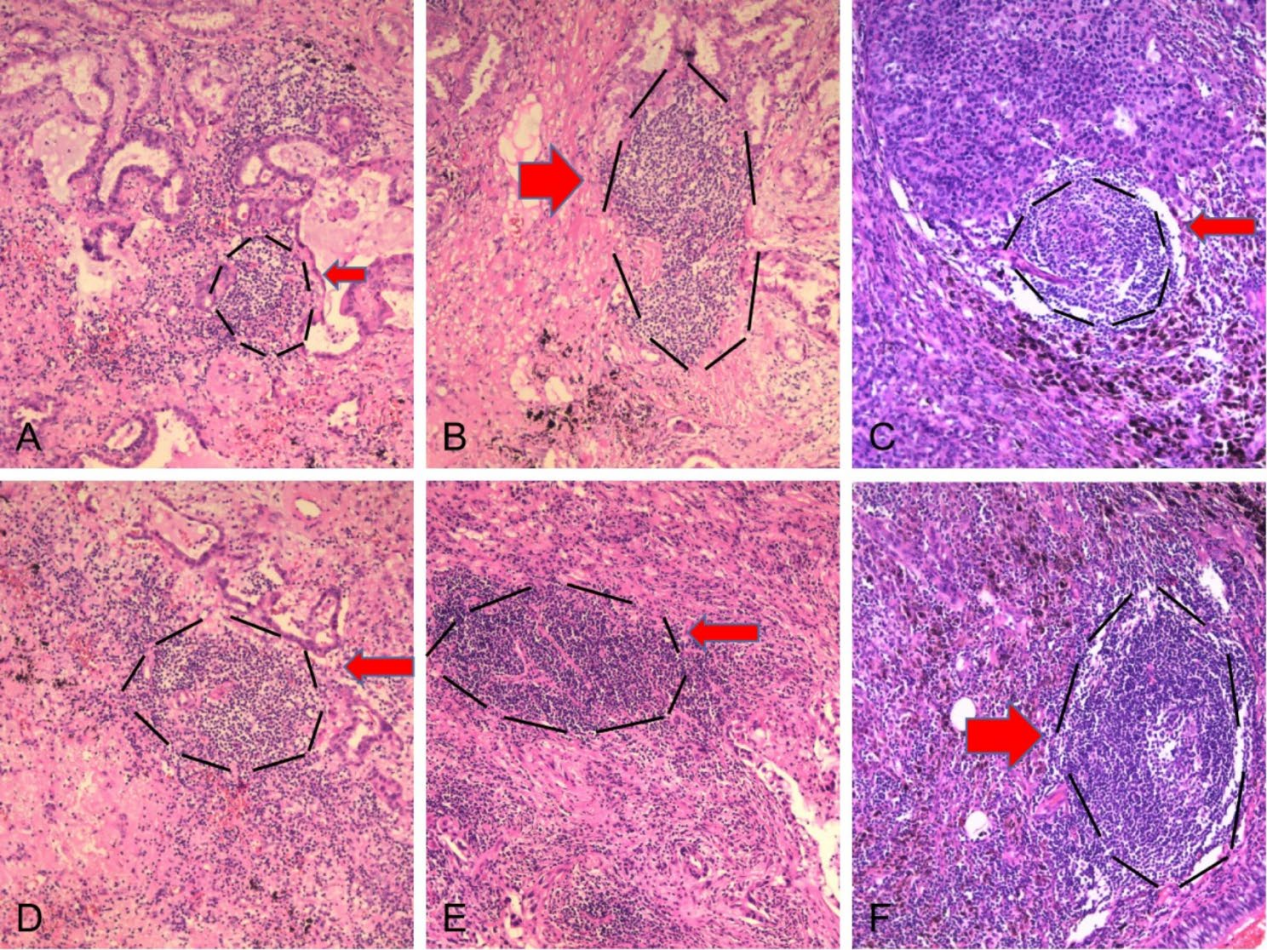
Different maturity and location of NSCLC-associated TLSs. (**A**) Agg (CT). (**B**) FOL-I (CT).(**C**) FOL-II (CT). (**D**) Agg (IM). (**E**) FOL-I (IM).(**F**) FOL-II (IM) .(x100)

TLS maturity and location were evaluated in H&E-stained sections from 151 patients with NSCLC. The proportion of patients with different TLS maturity in CT was 83.44% for Agg, 54.30% for FOL-I, and 15.23% for FOL-II. The proportion of patients with different TLS maturity at the tumor IM was 60.93% for Agg, 41.72% for FOL-I, and 10.60% for FOL-II (Fig. 2). Overall TLS, Agg (CT) and FOL-I (CT) were positively correlated with distant metastasis, UICC stage, and CD8 staining intensity in NSCLC patients. Agg (IM) was positively correlated with distant metastasis and UICC stage. FOL-I (IM) was positively correlated with UICC stage. FOL-II (IM) was positively correlated with distant metastasis. In addition, Agg (CT) was positively correlated with tumor differentiation. FOL-I (CT) and TLS (overall) were positively correlated with smoking status (*P* < 0.05, Table 2).

**Table 2:** Correlation between different TLS maturity and clinicopathological features in patients with NSCLC

Clinicopathological features	Cases		TLS(CT)*									TLS(IM)*									TLS*	
			Agg			FOL-I			FOL-II			Agg			FOL-I			FOL-II			(Overall)	
		Low	High	P	Low	High	P	Low	High	P	Low	High	P	Low	High	P	Low	High	P	Low	High	P
**Gender**				0.45			0.133			0.742			0.952			0.497			0.958			0.834
Female	84	51	33		59	25		70	14		55	29		51	33		75	9		60	24	
Male	67	44	23		50	17		57	10		40	27		37	30		60	7		46	21	
**Ages**				0.983			0.308			0.221			0.42			0.811			0.862			0.945
<60	63	39	24		48	15		52	11		40	23		36	27		56	7		44	19	
≥ 60	88	56	32		61	27		75	13		55	33		52	36		79	9		62	26	
**Smoking status**				0.272			0.037			0.974			0.923			0.053			0.05			0.006
Non-smoking	87	60	27		69	18		75	12		53	34		54	33		81	6		67	20	
Smoking	51	25	26		29	22		39	12		30	21		23	28		42	9		27	24	
**Differentiation**				0.04			0.119			0.879			0.642			0.658			0.232			0.466
Higher differentiation	99	60	39		68	31		81	18		62	37		58	41		86	13		68	31	
Middle -low differentiation	35	24	11		28	7		31	4		21	14		22	13		33	2		26	9	
**Histological type**				0.539			0.672			0.439			0.543			0.583			0.949			0.974
Adenocarcinoma	141	88	53		101	40		117	24		89	52		83	58		126	15		99	42	
Non-adenocarcinoma	10	7	3		8	2		10	0		6	4		5	5		9	1		7	3	
**Metastasis status**				0			0.001			0.139			0			0.154			0.031			0
Non-metastasis	56	24	32		30	26		39	17		25	31		28	28		46	10		28	28	
Metastasis	92	68	24		76	16		85	7		67	25		57	35		86	6		75	17	
**UICC stage**				0			0.008			0.13			0			0.028			0.119			0
I	47	18	29		23	24		30	17		18	29		22	25		38	9		20	27	
II	14	8	6		8	6		13	1		10	4		10	4		13	1		11	3	
III	57	37	20		45	12		53	4		37	20		31	26		52	5		43	14	
IV	32	31	1		32	0		30	2		29	3		24	9		31	1		31	1	
**CD8**				0.001			0.025			0.089			0.175			0.088			0.31			0.013
Negative	59	45	14		51	8		53	6		42	17		40	19		55	4		48	11	
Positive	84	45	39		54	30		67	17		49	35		45	39		74	10		53	31	

^*^TLS(CT):theTLS percentage of different maturity in the central tumor area

^*^TLS(IM): the TLS percentage of different maturity in the invasive margin area

^*^TLS(Overall): the TLS percentage of all maturity in the both central tumor and invasive margin area

### Prognostic significance of TLS at different maturity levels in CT and IM areas of NSCLC

**Fig. 3:**
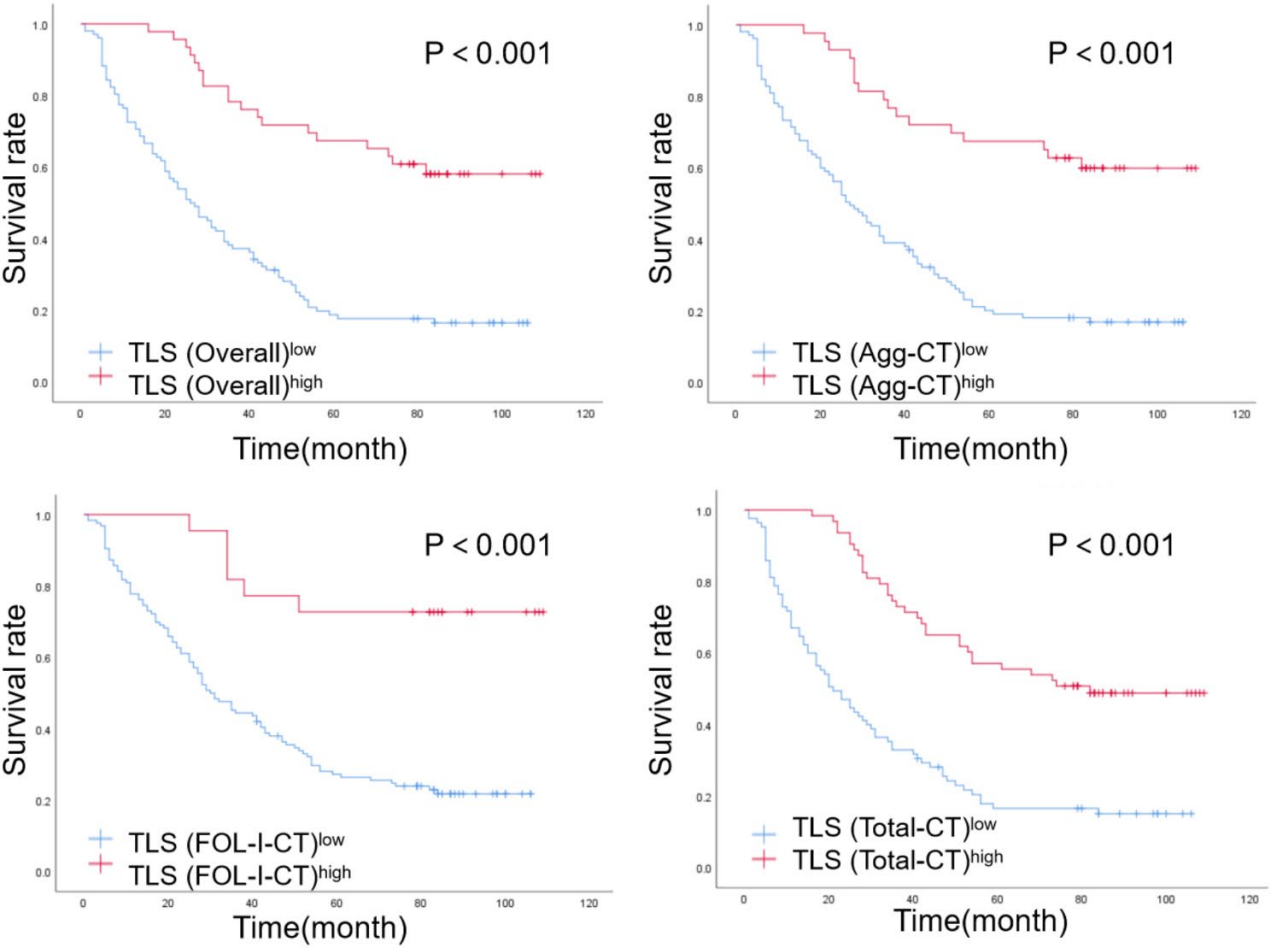
Kaplan–Meier survival curves of the TLS-high and TLS-low groups. TLS density correlated with patient OS outcomes

The median follow-up time from diagnosis was 64.5 months. Kaplan–Meier survival analysis showed that the average OS of the TLS-high (overall) group was 68.43 months, whereas the average OS of the TLS-low (overall) group was 35.79 months. The difference between the two groups was statistically significant (χ^2^ = 29.614, *P* < 0.001, Fig. 3). Univariate Cox regression analysis showed that gender, age, smoking status, and histological type of patients were not related to prognosis. Metastasis status, UICC stage, and different TLS maturity were correlated with patient prognosis. Subsequently, the preceding prognostic factors were included in the multivariate Cox regression analysis equation. The independent prognostic factors with risk effects were UICC stage and metastasis status, while those with protective effects were Agg (CT), FOL-I (CT), CTtotal, and overall TLS (*P* < 0.05, Table 3).

**Table 3:** Univariate and multivariate analysis of prognosis for patients with NSCLC

Clinicopathological features	Univariate analysis		Multivariate analysis	
	Hazard ratio (HR) (95% CI)	*P*	HR (95% CI)	*P*
Differentiation	2.056 (1.318–3.209)	0.002	1.516 (0.963–2.387)	0.073
UICC stage	16.236 (9.592–27.281)	< 0.001	7.416 (4.218–13.038)	< 0.001
**Metastasis**	12.001 (6.689–21.532)	< 0.001	8.819 (4.540–17.134)	< 0.001
Agg (CT)	0.272 (0.161–0.460)	< 0.001	0.401 (0.217–0.739)	0.003
FOL-I (CT)	0.212 (0.093–0.485)	< 0.001	0.267 (0.095–0.749)	0.012
FOL-II (CT)	0.226 (0.056–0.917)	0.007	0.267 (0.063–1.125)	0.072
Total (CT)*	0.319 (0.209–0.487)	< 0.001	0.560 (0.335–0.936)	0.027
Agg (IM)	0.335 (0.226–0.497)	< 0.001	0.762 (0.469–1.236)	0.271
FOL-I (IM)	0.624 (0.419–0.931)	0.021	0.774 (0.494–1.214)	0.265
FOL-II (IM)	0.432 (0.201–0.932)	0.032	0.699 (0.314–1.553)	0.379
Total (IM)*	0.356 (0.226–0.561)	< 0.001	0.640 (0.383–1.068)	0.088
Overall (CT + IM)*	0.273 (0.165–0.451)	< 0.001	0.435 (0.246–0.769)	0.004

^*^Total (CT):the TLS percentage of all maturity in the central tumor area

^*^Total (IM): the TLS percentage of all maturity in the invasive margin area

## Discussion

In malignant tumors, cancer cells maintain continuous cell growth by interacting with the host immune system and surrounding stromal cells. Immune checkpoint inhibitors are now part of standard therapy for advanced or recurrent NSCLC. Currently known predictors of NSCLC include programmed cell death ligand 1 expression in tumor tissue, tumor mutation burden, tumor-infiltrating lymphocytes, gut microbiota, and TLS [9], which forms new opportunities for cancer therapy. TLS may be a new predictive biomarker for immune checkpoint blocking. TLS reflects the tissue aggregation of immune cells in non-lymphoid tissues formed after birth. Under normal physiological conditions, TLSs are not present in the body. They start appearing during chronic inflammation, such as autoimmune diseases, chronic infections, and cancer. In malignant tumors, mature TLSs induce antitumor responses [10]. TLS-associated dendritic cells are key to establishing local T-cell-mediated antitumor responses by presenting tumor antigen peptides to T cells located in T cell TLS compartments [11]. Successful binding between naïve CD8^+^ T cells and antigen-presenting cells activates immature T cells into CD8^+^ T cells with cytotoxic functions, which can kill intracellular pathogens and tumor cells. However, B cells in immature TLSs may produce inhibitory factors that inhibit immune cell function. Overall, TLSs have a bivalent influence on host–tumor interactions, and they are found in almost all solid tumors. Studies have shown that TLSs indicate a favorable prognosis for most human tumors, including breast cancer [12], lung adenocarcinoma (LUAD) [13], hepatocellular carcinoma [14], colorectal cancer [15], cutaneous squamous cell carcinoma [16], gastric cancer [17], cervical cancer [18], and oral cancer [19].

The significance of TLSs in lung tissue has raised concerns. Hong et al. used clinical data from 515 LUAD patients in a The Cancer Genome Atlas (TGCA) cohort to investigate the association between TLS markers and the immune microenvironment, tumor mutation burden, and driver gene mutations. They showed that LUAD patients with high TLS characteristics had good immune microenvironment and good prognosis [11]. Karīna et al. used immunohistochemistry to show that TLS density was the most important independent prognostic marker in untreated LSCC patients, and its performance was superior to tumor stage [20]. Mehrdad et al. quantified TLS in resected NSCLC whole-tumor tissue sections by considering the ‘absolute count’ of Agg and/or FOL [21]. Only a few studies have examined TLS maturity in LUAD. Ren et al. found that TLS maturity was higher in patients with higher tumor stages [22]. Wakasu et al. suggested that mature TLSs may support antitumor immunity through lymphocyte activation [23].

This study examined the associations between TLS and OS, patient pathologic characteristics, and CD8 staining intensity. This study revealed that CTtotal and overall TLS in NSCLC tissue were independent prognostic factors for good patient prognosis, consistent with the results of previous studies [21, 24]. However, most studies only examined TLS in tumor tissue according to density and location. To the best of our knowledge, this study is the first to identify immature CT TLSs [Agg (CT) and FOL-I (CT)] associations with clinicopathologic features and as independent prognostic factors associated with good prognosis and protective effect. These results are advantageous, because the area ratios of Agg (CT) and FOL-I (CT) in H&E sections are easier to quantitate than other indicators, and only the CT part requires investigation. A relatively large specimen tumor volume often cannot be retrieved completely in pathological sampling. The important role of indicators in the CT region in NSCLC can help patients with large tumor volume (especially advanced stage patients). TLS can be evaluated as a diagnostic H&E component and can be easily introduced as a relevant prognostic parameter in routine pathology.

Moreover, this study revealed that FOL-I (CT) (*P* = 0.037) and overall TLS (*P* = 0.037) were related to smoking status in NSCLC patients. Mathieu et al. investigated the mechanisms of tertiary lymphoid tissue (TLT) formation in the lungs of cigarette smoke-exposed mice [25]. Cigarette smoke-induced pulmonary TLTs included T cells, B cells, dendritic cells, and macrophages. This finding suggests that lung inflammation caused by smoking contributes to TLS formation.

Considering the study sample, the limitations of this study include the low number of cases and the retrospective single-center evaluation. However, the sample included a large number of advanced patients, consistent with the main beneficiaries of this study. Further research is required in depth and exploit the underlying mechanism.

In conclusion, the presence of different TLS maturity and location provides new insights into prognosis for NSCLC patients. Immature CT TLS can be a crucial clinicopathological parameter.

## Data Availability

The datasets used and analysed during the current study are available from the corresponding author on reasonable request.

## Abbreviations

Agg: aggregates
CI: confidence interval
CT: central tumor
FOL: follicles
H&E: hematoxylin–eosin
HR: hazard ratio
IM: invasive margin
IM: invasion margin
LSCC: lung squamous cell carcinoma
LUAD: lung adenocarcinoma
NSCLC: non-small cell lung cancer
OS: overall survival
SCLC: small cell lung cancer
TLS: tertiary lymphoid structure
TLT: tertiary lymphoid tissue
UICC: Union for International Cancer Control
